# Supplementary figures and images for: Pharmacological and pharmacokinetic profile of the novel ocular hypotensive prodrug CKLP1 in Dutch-belted pigmented rabbits
Source: PLoS One. 2020 Apr 16;15(4):e0231841. doi: 10.1371/journal.pone.0231841 (PMC7162492; doi:10.1371/journal.pone.0231841)

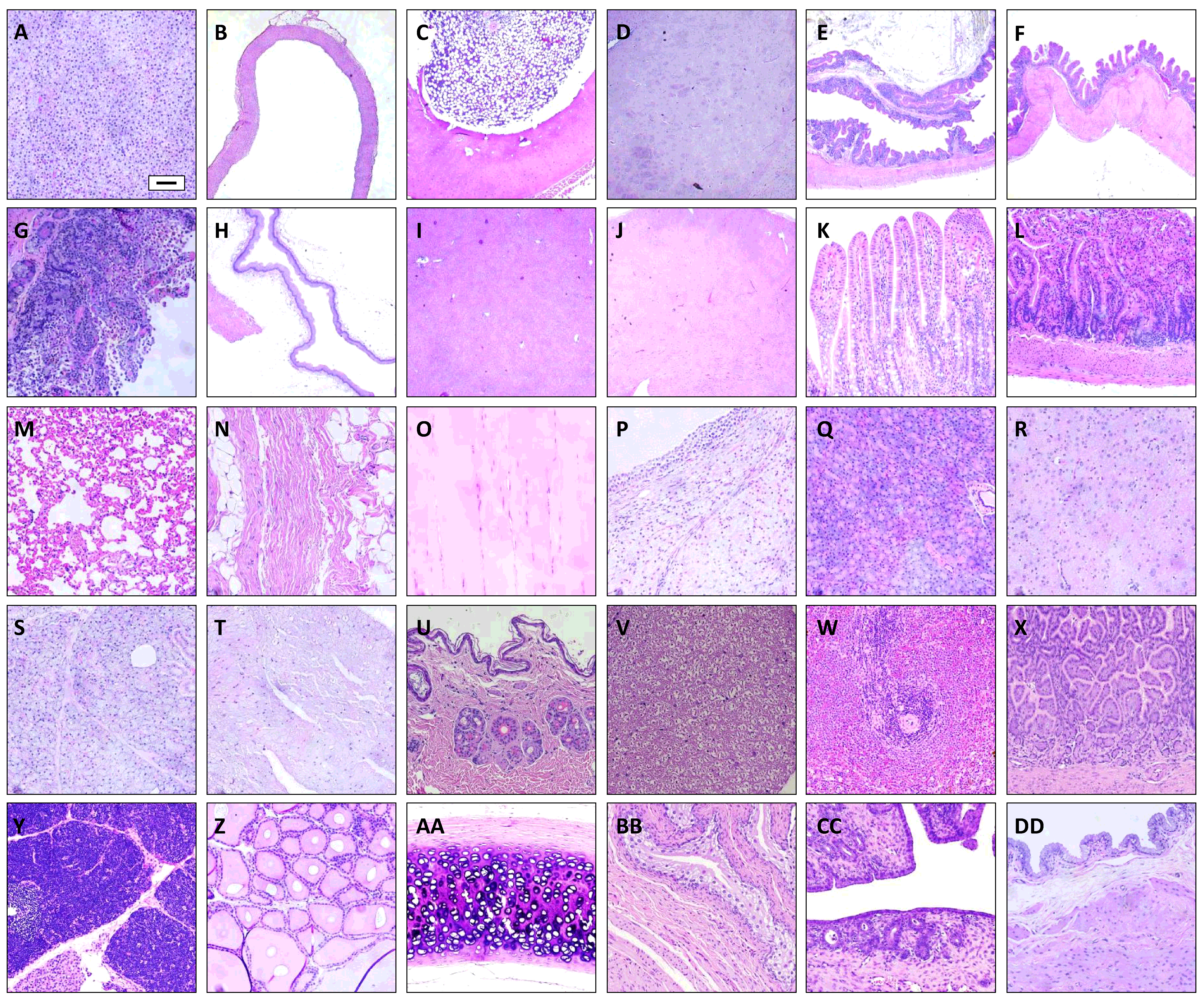

Supplement: S1 Fig — No observable differences in histology were noted between vehicle-treated and CKLP1 treated tissues as evaluated by a masked veterinary pathologist following 90 days of treatment with CKLP1 eye drops. A, adrenal gland; B, Aorta; C, bone marrow; D, brain; E, cecum; F, colon; G, duodenum; H, esophagus; I, gall bladder; J, heart; K, ileum; L, jejunum; M, lung; N, mammary gland; O, muscle; P, ovary; Q, pancreas; R, pituitary gland; S, salivary gland; T, sciatic nerve; U, skin; V, spinal cord; W, spleen; X, stomach; Y, thymus; Z, thyroid; AA, trachea; BB, urinary bladder; CC, uterus; DD, vagina. Scale bar, 50 μm. (TIF) [file pone.0231841.s001.tif]
