# Supplementary material for: Pharmacological and pharmacokinetic profile of the novel ocular hypotensive prodrug CKLP1 in Dutch-belted pigmented rabbits
Source: PLoS One. 2020 Apr 16;15(4):e0231841. doi: 10.1371/journal.pone.0231841 (PMC7162492; doi:10.1371/journal.pone.0231841)
Supplement: Attachment — Submitted filename: Response to reviewers comments-PONE-D-20-00899.docx [file pone.0231841.s002.docx]

We thank the reviewers for taking the time to carefully review our manuscript. Based on their comments, we have made changes to the document and have addressed their concerns. We believe that the manuscript is much improved due to the insightful comments of the reviewers. Please find below each reviewer’s comment (black text) and our response (blue text)

Reviewer #1

1. In Figure 2 the authors reference the half-life CKLP1 and Levcromakalim. Can this be highlighted better in the figure? The number is referenced in the text but it would be useful to denote the half-life on the graph as well.

We have added the half-life of CKLP1 and levcromakalim in bottom right corner of each panel in figure 2.

2. Lines 191-192 references Figure 3 and the peak concentration comparisons between days. Can this data be statistically compared?

As per the reviewer’s suggestion, we compared the peak concentration values from all samples (n=3 animals for each day) using one-way ANOVA. The resulting p value was not statistically significant (p=0.47). This has been added to the text (line 212). Note that no post hoc tests were performed due to non-significant p value. We also added the following text to the discussion (lines 324-331) – “It should also be noted that various pharmacokinetic parameters such as higher peak CKLP1 and levcromakalim concentrations at days 4 and 8 compared to day 1, as well as the amount of levcromakalim detected in the ocular fluids, were not statistically significant. This is not surprising since the current study was not designed to determine significance across days but to evaluate if there were any trends. These results provide us with the level of differences in drug concentration we can expect following repeated treatments. Experiments designed to test accumulation of drugs in ocular fluids and tissues will require a larger study that is correctly powered.”

3. Figure 4, Can this data also be statistically compared (Line 214)?

As suggested by the reviewer, we ran statistics on levcromakalim concentrations in aqueous and vitreous humor for both right and left eyes. We did not find any statistical significance for levcromakalim concentrations in aqueous or vitreous. This has now been added in the Results section (line 230-232). We have also added a “Statistical analysis” section to methods, describing the general statistical methods applied (lines 157-163). Exclusion of this section in the original manuscript was an oversight on our part and we thank the reviewer for raising this concern.

4. Provide more detailed methods on how IOP was measured and what statistical analysis was performed on these data.

The methods section has been updated with a brief description of IOP measuring protocols (lines 121-125). We have also added a reference which describes the IOP measurement protocol in more detail (Roy Chowdhury et al. 2017, IOVS, 58(13), p 5731-41). In regards to statistics, we have now included a new section titled “Statistical analysis”, under methods describing the general statistical analysis applied (lines 157-163).

Reviewer #2:

1. The authors note that IOP is reduced by 2.4 +/- 0.4 mm Hg and 14.1 +/- 1% compared to the other eye of the same rabbit. While this is nice, what was the IOP compared to the baseline IOP in the eye that was actually treated? In other words, what was the magnitude and percent of IOP reduction in the eye that was treated, pre- and post-? Getting data from both eyes (pre- and post- drug or vehicle) controls for more confounders.

Treatment with CKLP1 lowered IOP by 11.4% when compared to baseline. Following cessation of treatment there was no statistical difference between pre- and post-treatment IOP. We have also clarified in the methods (line 152), results (lines 243-247) and in discussion (lines 335-337) that following termination of treatment, three rabbits were sacrificed 24 hours after last dose and in the remaining three rabbits, IOP was measured daily for 3 days for calculation of post-treatment IOP. As a result the post-treatment IOP values are from only three animals.

2. While the C_max_ for CKLP1 and levcromakalim may not have been significantly different in the 1- to 8-day experiments (Table 2), it appears that the Tmax might be? Do that calculation and include/implement. It is interesting to me because it says that the loading is faster. Makes sense given that the drop has been there for 7 days prior. The AUCs too.

As per the reviewer’s suggestion, we ran one-way ANOVA for the various pharmacokinetic parameters (C_max_, T_max_, AUC) that appear to present different values across the three days (1, 4 and 8). None of the parameters showed statistically significant changes between days 1, 4 and 8. We have updated the text in the results section accordingly (lines 214-216). This is most likely due to our small sample size. To clearly evaluate the pharmacokinetic trends across doses and regimens, a much larger study with a broader scope of data analytics will be required. Text describing this has also been added to the discussion section (lines 324-331).

3. If cromakalim (which is broken down to a dex- and lev-type) can lower IOP (citation 13), what is the advantage or need for CKLP1? Because it is already the lev-type? If one understood the breakdown of cromakalim and ratio of dex- and lev- that is produced, all one has to do is to upscale the dose of cromakalim to get more lev-. Further, the dex- had some IOP lowering effect too (citation 13). Unless, the dex- has some potential side-effect?

Commercially available cromakalim contains both the dex and lev forms, although levcromakalim is the main chiral component. Cromakalim, both dex and lev, are insoluble in water and dissolve only in organic solvents such as DMSO, rendering it unsuitable for application to human eyes. In order to overcome this limitation, we developed CKLP1 as a water soluble prodrug of levcromakalim and characterized its ocular hypotensive activity. The current study is the initial evaluation of pharmacokinetic properties of CKLP1 in order to facilitate its development for human trials. [Note: For additional details on the development of CKLP1, the reviewer is referred to our previous publication (Roy Chowdhury U, Viker KB, Stoltz KL, et al. J Med Chem. 2016;59(13):6221-31)].

4. For the topical experiments, it is slightly strange that absolutely no CKLP1 is found in the aqueous and vit. The authors suggest that testing the levels 24 hours after the last drop may have caused the aqueous turnover to make the drug leave the eyes (lines 285-298). I have a few thoughts.

-First, topical drugs have a much more direct route to the systemic body than aqueous outflow. Topical drugs simply enter the nasolacrimal duct system (like how we taste our salty tears when we cry). The drug can then be picked up by the blood vessels in the oropharynx and nasal mucosal epithelium. This alone can explain presence of drugs in the blood stream after topical delivery in the short-term experiments. This is also an issue for comment #5 below.
-Second, maybe the drug does not even have to get into the eye. The authors maybe testing the aqueous and vitreous under the assumption that that drugs must move into the eye to impact IOP-governing mechanisms. However, as the authors note, these drugs are supposed to affect the distal outflow pathways (lines 59-60). Thus, could the drug just penetrate into the scleral tissue and directly impact outflow as opposed to entering the anterior chamber first to do so?
-Third, the methods involve taking the aqueous/vit/plasma, spinning it down and testing the free fractions. What if CKLP1 is pigment bound? Beta blockers can be. In this case, the iris may have absorbed it all. Can we test for drug in iris? Try homogenization iris followed by a fast spin and testing what is in the sup.
-Fourth, what if there is differential protein binding such that the drug in the vitreous cavity was actually stuck with the vitreous that had not undergone syneresis? To address this, the pellet could be collagenase treated and spun again with the sup tested.
-Lastly, maybe the phosphatases are so fast that all the CKLP1 was metabolized.

We agree with all the potential scenarios laid out by the reviewer. We have tried to add some of our own thoughts in response to the insightful comments of the reviewer.

1. The reviewer is correct and we agree that this is one way the drug may be entering the blood stream. We have mentioned this in the manuscript (lines 315-316).
2. A scleral route of drug entry and localized action is quite possible. However, in previous studies, we have noticed molecular changes in cells of the trabecular meshwork in response to drug treatment in vivo (Roy Chowdhury U, Bahler CK, Holman BH, Fautsch MP, PLOS ONE, 2017, 12, e0179345). Furthermore, cromakalim also lowers pressure in perfusion cultured human anterior segments. These results indicate that in addition to a possible scleral route, drug entry through the trabecular meshwork is also feasible.
3. The reviewer raises a very interesting point. We are actively looking at tissue and cellular compartments inside the eye which can act as a local reservoir of CKLP1 and/or levcromakalim. We are in the final stages of collating data from a dog study where we are examining some of these questions. The drug has been licensed by a startup company and many of these experimental designs the reviewer is referencing are being performed in preparation for an IND submission. Currently, we do not have access to this data.
4. Another excellent thought. Unfortunately, at the time of collecting the tissues, we had no way of knowing the kind of results we would obtain, including the difficulty of pin pointing the exact metabolic profile of CKLP1 in the studied tissues. However, we are in the process of collecting data from dog studies that may help us determine if there are any ocular reservoirs involved in drug storage.
5. It is certainly a possibility that the phosphatases are cleaving all of CKLP1. However, we have seen just the opposite in vitro (unpublished data), that CKLP1 is never completely cleaved by phosphatases to cromakalim. Understanding this mechanism of CKLP1 to cromakalim conversion will require an extensive experimental design, which is beyond the scope of this study.
5. Then for the topical experiments, it is strange that after 90 days, no levels were detected in the plasma. This is quite strange because both drugs were detected systemically after the shorter experiments at the 24-hour time-point. Further in 2 of 6 animals, no levcromakalim was seen in the aqueous while in 3 of 6 animals no levcromakalim was seen in the vitreous. This was unlike the shorter topical experiments were levcromakalim was more consistently detectable. Thus, the drug has to be somewhere. The renal/liver data suggests some elimination mechanism but this seems not enough. Since the drops applied on day 88 and 89 aren’t enough to show drug in the plasma, either the clearance method is revved up or there a depot where the drug is just accumulating. My feeling is that the drug is going somewhere that we are not recognizing. As above, I would look for the drug in other ocular tissues like the actual vitreous itself (you are only testing the liquefied portion) and the iris. Also, in the blood fraction, test for binding. One could repeat the 90-day experiment, collect the blood and test for the drug in the packed RBC portion (after the spin). Alternatively, just test for in vitro binding by getting rabbit whole blood, mixing it with drug, waiting for a period of time, spinning the blood down and testing for the drug in the plasma vs. packed RPC portion. At least, this in vitro experiment could show if the drug can get into or onto RBCs and then help solve some of the mystery.

   It is interesting that we do not detect the drug in plasma after 90 days. While we would like to think that it is efficiently excreted, we have no data conclusively supporting this. We were not able to measure CKLP1 or levcromakalim levels in urine by mass spec. We believe this may be due to masking of the drugs by secretory lipids in order to facilitate excretion. In reference to RBCs, we do not have any indication that CKLP1 binds to metallo-proteins like hemoglobin. We do believe that a more global approach is required to understand the entire tissue localizing profile of CKLP1 and its metabolites including levcromakalim (lines 329-331).

However, we can explain why levcromakalim was seen in only 3 out of the 6 animals. For the pharmacokinetic studies following topical application, we sacrificed 3 animals 24 h after the last dose and allowed a washout period of 3 days for the remaining 3 animals. The 3 animals where the drug was identified in aqueous and vitreous were from the 3 animals sacrificed after 24 hr. following last dose. Only 1 animal of the washout group showed levcromakalim in aqueous humor. No drug was identified in the vitreous of this animal and in the aqueous and vitreous of the other 2 animals that were allowed a washout for 72 hr. The reason behind doing a washout period was so we could obtain IOP data and determine how long it took to return to baseline. This has now been clarified in the results and discussion sections (lines 243-247, lines 334-337).

Minor issues:
1) lines 139… for this section, you must mean “for rabbits treated with CKLP1 for up to 8 days..” as opposed to just 8 days since some rabbits received treatment for 1- and 4-days.

Thank you for pointing this out. The text has been changed (line 143).

2) lines 301: levcromakalim cannot be “consistently” identified if it is only seen in 3 of 6 rabbits for the vitreous and in 4 of 6 rabbits in the aqueous.

We have taken out “consistently” but also modified the sentence with the added information that levcromakalim was identified in all rabbits sacrificed at 24 hours after treatment (lines 334-36 in the revised manuscript).
